# Supplementary material for: Accounts of preventative coping: an interview study of stroke survivors on general practice registers
Source: BMJ Open. 2022 Sep 5;12(9):e058441. doi: 10.1136/bmjopen-2021-058441 (PMC9445822; doi:10.1136/bmjopen-2021-058441)
Supplement: Author's manuscript [file bmjopen-2021-058441.draft_revisions.pdf]

BMJ Open is committed to open peer review. As part of this commitment we make the peer review history of every article we publish publicly available.

When an article is published we post the peer reviewers' comments and the authors' responses online. We also post the versions of the paper that were used during peer review. These are the versions that the peer review comments apply to.

The versions of the paper that follow are the versions that were submitted during the peer review process. They are not the versions of record or the final published versions. They should not be cited or distributed as the published version of this manuscript.

BMJ Open is an open access journal and the full, final, typeset and author-corrected version of record of the manuscript is available on our site with no access controls, subscription charges or pay-per-view fees (http://bmjopen.bmj.com).

If you have any questions on BMJ Open's open peer review process please email info.bmjopen@bmj.com

BMJ Open

Stroke survivors' accounts of preventative coping: An interview study

Journal:	*BMJ Open*
Manuscript ID	bmjopen-2021-058441
Article Type:	Original research
Date Submitted by the Author:	24-Oct-2021
Complete List of Authors:	Scott Reid, Peter; University of Cambridge Primary Care Unit<br>Neville, Emma; University of Cambridge Primary Care Unit<br>Cater, Frances; University of Cambridge Primary Care Unit<br>Mullis, Ricky; University of Cambridge, Department of Public Health and Primary Care<br>Mant, Jonathan; University of Cambridge, General Practice and Primary Care Research Unit<br>Duschinsky, Robbie; University of Cambridge Primary Care Unit
Keywords:	Stroke < NEUROLOGY, CARDIOLOGY, REHABILITATION MEDICINE

SCHOLARONE™
Manuscripts

I, the Submitting Author has the right to grant and does grant on behalf of all authors of the Work (as defined in the below author licence), an exclusive licence and/or a non-exclusive licence for contributions from authors who are: i) UK Crown employees; ii) where BMJ has agreed a CC-BY licence shall apply, and/or iii) in accordance with the terms applicable for US Federal Government officers or employees acting as part of their official duties; on a worldwide, perpetual, irrevocable, royalty-free basis to BMJ Publishing Group Ltd ("BMJ") its licensees and where the relevant Journal is co-owned by BMJ to the co-owners of the Journal, to publish the Work in this journal and any other BMJ products and to exploit all rights, as set out in our licence.

The Submitting Author accepts and understands that any supply made under these terms is made by BMJ to the Submitting Author unless you are acting as an employee on behalf of your employer or a postgraduate student of an affiliated institution which is paying any applicable article publishing charge ("APC") for Open Access articles. Where the Submitting Author wishes to make the Work available on an Open Access basis (and intends to pay the relevant APC), the terms of reuse of such Open Access shall be governed by a Creative Commons licence – details of these licences and which Creative Commons licence will apply to this Work are set out in our licence referred to above.

Other than as permitted in any relevant BMJ Author's Self Archiving Policies, I confirm this Work has not been accepted for publication elsewhere, is not being considered for publication elsewhere and does not duplicate material already published. I confirm all authors consent to publication of this Work and authorise the granting of this licence.

For peer review only

Stroke survivors' accounts of preventative coping: An interview study

For peer review only

Author and Co-author's name: Peter Scott Reid, Emma Neville, Frances Cater, Ricky Mullis, Jonathan Mant & Robbie Duschinsky

Corresponding author: Robbie Duschinsky, East Forvie Site, University of Cambridge, Cambridge, CB2 0PY, 01223 746426
rd522@medschl.cam.ac.uk

Affiliation all authors: Primary Care Unit, University of Cambridge, East Forvie Site, Cambridge, CB2 0PY

Key words:
Chronic illness, coping, secondary prevention, self-concept, stroke, thematic analysis

Word count: 4017

For peer review only - http://bmjopen.bmj.com/site/about/guidelines.xhtml

Abstract

Objectives: Preventative coping is an underexplored aspect of coping behaviour. Specifically, coping is a key concern in stroke survivor accounts, but this has yet to be investigated with reference to secondary prevention.

Design: Based on a sample of stroke survivors identified through General Practice stroke lists, 21 semi-structured interviews were conducted to examine how stroke survivors characterise their coping process. The interviews were coded using thematic analysis.

Results: Two key themes were identified, which foregrounded the role of self-concept for coping: a) striving to be 'good', b) appeal to ideas of 'personality'.

Conclusions: We highlight that coping was characterised by survivors as something enacted by the individual self, and consider how constructions of self may impact preventative coping efforts.

For peer review only - http://bmjopen.bmj.com/site/about/guidelines.xhtml

Strengths and limitations

- The study drew upon detailed qualitative characterisation of the perceptions of stroke survivors of aspects of coping with reference to secondary prevention
- The analytic approach foregrounded both lines of consistency and variation in how stroke survivors spoke about their experiences
- The data relate to participants' perceptions of their coping, rather than their coping practices
- From a single interview with each participants, we cannot draw firm conclusions about how coping is embedded in aspects of participants lives over time.

Competing interests: N/A

Key words:
Chronic illness, coping, secondary prevention, self-concept, stroke, thematic analysis

For peer review only

Introduction

Mortality from stroke in the UK is declining but the prevalence of stroke is rising due to improved survival rates and an aging population.[1] Stroke frequently results in major life changes and can influence a person physically, psychologically and socially.[2,3] Stroke survivors living in the community often report they have little support – but also anticipate little support since they feel they are obliged to take personal responsibility for managing their own recovery.[4,5] For instance, Fletcher et al. found themes of self-responsibility and the power of medical authority in their study of stroke survivors.[6] They interpreted their findings in terms of Foucauldian ideas of power, in which survivors were enjoined to discipline themselves in order to effect a successful recovery, and relieve pressure on health and social care services. Commentators have suggested that this construction of stroke survivorship reflects broader discourses on chronic illness that highlight individual responsibility and self-sufficiency, under guidance from distant experts but with limited service provision.[7,8]

Understanding stroke survivors' perspectives through the lens of 'coping' can add knowledge of the needs of this patient group as well as inform services for stroke survivors. Perhaps the classic definition of coping was offered in the 1980s by Lazarus and Folkman, who characterise it as the "thoughts and behaviours that people use to manage the internal and external demands of situations that are appraised as stressful".[9] 'Coping' used in this way is

For peer review only - http://bmjopen.bmj.com/site/about/guidelines.xhtml

the attempt to manage challenges in life, rather than necessarily being successful in that attempt. Most work on coping has addressed the challenge of how individuals manage current problems. Yet in recent years scholarship has increasingly emphasised coping as frequently a joint undertaking.[10,11] Sometimes this is explicit, with actors aware of the coordinated or collective work 'we' do to cope. Sometimes this is implicit, with actors less able to willingly acknowledge the contribution of others to 'my' coping with the demands of situations. Such accounts have been frequently found to be shaped by perceptions of the source of the stressful demands, and on attributions about others, and the extent to which the demands are felt to be a disruption or in continuity with past experiences.[12]

Much qualitative research on stroke survivors has been focused on the immediate period up to one year following the stroke, thus prioritising the initial stages of disruption.[13,14,15,16,17] A few studies have conducted later follow-up, but with a focus on how survivors deal with the effects of the initial stroke and not on secondary prevention.[18,19] More recently, secondary prevention following stroke has seen attention by a small number of studies and reviews,[20,21,22] but these acknowledge that the topic remains underexplored. In particular, there has been relatively little attention to 'preventative coping', which can be defined as the

For peer review only - http://bmjopen.bmj.com/site/about/guidelines.xhtml

efforts individuals make to prepare for uncertain events in the long run by building up resources that will result in less strain in the future by minimising the event's severity.[23]

Guidelines emphasise the importance of changes to lifestyle and the role of medication in reducing the risk of having a second stroke.[24] While they stress the importance of a personalised approach, this is in terms of what to recommend rather than how to optimise chances that the recommendations will be adopted. Analysis of reasons for this sub-optimal use that explore patient perspectives have tended to focus on aspects such as self-care ability, knowledge and understanding, rather than the role of preventative coping strategies. Furthermore, what attention is given to preventative coping tends to treat it as the action of individuals, rather than a joint or jointly-coordinated activity with others. Better understanding of preventative coping strategies might lay the ground for more nuanced ways of developing approaches to supporting secondary prevention after stroke. This article therefore sets out to answer the question: how do stroke survivors characterise their coping process in the context of secondary prevention?

For peer review only - http://bmjopen.bmj.com/site/about/guidelines.xhtml

Methods

Data collection

A secondary analysis was conducted on transcripts of semi-structured interviews carried out to inform the design of an intervention aimed at improving primary care after stroke. Interviews took place February-May 2016. Sampling was conducted through stroke registers held by general practices in Cambridgeshire, Peterborough and Bedfordshire. If the register held more than 150 cases then patients were sorted randomly and then 150 extracted. A GP then screened the list to remove any patients that did not meet the inclusion criteria or who met exclusion criteria. Inclusion criteria were: 1) Confirmed diagnosis of stroke from survivor's records. 2) Has a good understanding of English. 3) Has the capacity to provide written informed consent. Exclusion criteria were: 1) Survivors considered by their GP to be sufficiently ill that it would make participation difficult. Expressive aphasia was not a basis for exclusion. 21 participants were interviewed (Table 1). The sample was made up of 11 men and 10 women ranging in age between 49 and 93 (mean 72.5). Interviews took place on average 6 years 5 months after stroke. Ethical permission for the study was given by the ethics committee at [University]. In conducting interview research with stroke survivors we considered carefully potential risks of participation, and worked to minimise these, for instance through being

For peer review only

rigorous in ensuring informed consent and that participants could exercise their right to withdraw.

Table 1 – Participant information

**Total subjects**	21
Men	11
Women	10
**Age**	
Range	49 to 93
Mean	72.5
Median	71
Standard Deviation	11.8
**Barthel Index as a measure of disability (Total score, out of 100)**	
Range	50 to 100
Mean	89.8
Median	100.0
**Length of time since stroke**	
Range	1 year 8 months to 22 years 6 months
Mean	6 years 5 months
Median	3 years 6 months
Standard Deviation	5 years 11 months

Interviews were conducted in participants' homes. An interview guide was used with questions focused on life after stroke. This included questions on family and social relations, the participants' sense of self, their emotions, interactions with health care professionals, and

For peer review only - http://bmjopen.bmj.com/site/about/guidelines.xhtml

For peer review only

how they reflected on their experiences. Questions addressed both the nature of small practical adjustments required and participants' wider sense of their experiences. Follow-up questions were used to probe for relevant details.

Public and Patient Involvement

A PPI member was involved in developing the research as a co-applicant. The results of the interviews were fed back to a multi-disciplinary intervention development group that included PPI representation who helped placed the results in context. The research was overseen by an independent steering committee that included a PPI member. The open ended questions used in the interviews were informed by meetings with community stroke survivor and carer support groups.

Thematic analysis

Thematic Analysis (TA) is a "technique for identifying, analysing and reporting patterns (themes) within data".[25] Researchers using TA have praised its capacity to tease out contradictions between statements saying one thing and statements suggesting another, and to explore how people construct and give account of their attitudes.[26] TA was combined with a constant comparative approach, using existing theory and literature from coping and qualitative work on life after stroke to sensitise our development of codes, theme generation, and analysis.

For peer review only - http://bmjopen.bmj.com/site/about/guidelines.xhtml

For peer review only

Two co-authors collaborated on the development of the codebook for the TA, which contained the code, a definition, guidelines for when and when not to use the code, and examples.[27] The first author then coded all of the verbatim interviews using NVivo software. Themes were identified as aggregates of codes, speaking to broader ideas in the literature. These themes were revised and refined several times by looking at how they held up as valid interpretations of the data when referring back to the transcripts, and on the basis of discussion with the rest of the study team.[28]

Findings

Participants all spoke about their preventative coping efforts. Some employed the word 'coping' itself while others described how they evaluated and dealt with the challenges of secondary prevention using different terms. What was most striking about the accounts was the strong emphasis of participants on individual efforts. There was little explicit acknowledgement that coping might be a jointly pursued activity, though this was implicit. Rather, the two most prominent themes with respect to secondary prevention were a) the need for the self to strive to be 'good', and b) the role of 'personality' as a resource for these efforts.

For peer review only - http://bmjopen.bmj.com/site/about/guidelines.xhtml

Striving to be 'good'

Across the interviews, participants employed the language of discipline: 'don't eat silly things', 'strict', 'moderation', 'it's me own fault', 'control', 'careful'. This underwrote their depictions of preventative behaviour in moralistic terms, and the weight of expectation they felt to be 'good'. Participants often discussed how doctors had evaluated their efforts to change their health-affecting behaviours and habits such as eating, drinking, and exercise. One interviewee relayed the advice given to her about losing weight while coupling it with the doctor's approval of her habits and behaviours:

> They did say to me "Try and lose a little bit of weight", but you know I mean he came, the consultant came in on the day I came home and he said "This is where I should be saying to you stop smoking, stop drinking, there's your cholesterol tablets and there's your blood pressure tablets" and he said "I can't" because I don't smoke, I've never smoked, the occasional drink and the other two were fine so he said "You know, try and get a little bit of weight off and just have your exercise," and yeah. (Woman)

The participant here depicts their successful prevention efforts, constructed in contrast to the hypothetical and less adherent patient that her doctor described. Another patient described interactions with his doctor about changing his drinking behaviour to better prevent a stroke:

> I wasn't happy with the doctor I saw, she kept telling me oh you can have another stroke very quickly, you know. Yeah you'll soon have another stroke if you don't do this and you don't do that. If you do too much you'll have another stroke, you know that don't you. If you do…and this was continual, this woman kept telling me this. I do drink more than I should do, I admit that, not a vast amount, but I do drink more than I should do. I drink

For peer review only - http://bmjopen.bmj.com/site/about/guidelines.xhtml

wine, and I do enjoy a nice drink. But she said oh, if you have…drink, you know, more than one glass full you're going to have a stroke. (Man)

The two accounts above, as with most of our interviews, depicted doctors as external adjudicators of individuals' coping efforts. Though one (woman) speaks of broadly being affirmed and the other (man) of feeling chastised for a perceived lack of effort, they both draw on doctors as arbitrating the validity of their attempts to prevent a future stroke and how 'good' they have been. In contrast to the prominent role given to doctors, other healthcare professionals and family members were mentioned in other parts of the interview but not generally in discussions of secondary prevention.

One curious aspect of the accounts was that, despite commonalities in the language of discipline used, survivors did not necessarily give the same description of what being 'good' or 'bad' entailed. Participants were variously concerned with striving to be good in their medication, smoking, weight, exercise, or alcohol use – or some combination of the above, though very rarely with more than a few. What the accounts had in common was the conception that participants felt that they should be *striving* for these changes or behaviours – even if they currently fall short or disagree with their doctor's judgement of success. This theme suggests that participants construct and use categories of 'good' and 'bad' behaviour in relation to

For peer review only - http://bmjopen.bmj.com/site/about/guidelines.xhtml

prevention, and that moralistic terms are even used by those survivors feeling they have behaved in ways that are not 'good'.

Appeal to personality

A consistent theme throughout the interviews was survivors' appeal to the idea of 'personality' or more generally stable traits and qualities in accounting for their coping efforts. Participants consistently described the self as fixed rather than a malleable set of traits. They perceived this stability as of fundamental importance in hindering or helping their preventative coping attempts, and in explaining how their experiences prior to stroke shaped the way they dealt with the challenges they faced. One of the women in the sample was asked how she had coped emotionally since the stroke:

> I'm not a very emotional person, I don't think, really. I mean, I just think things in my head, but I never cry, never, ever cry. I don't know why, I think that's something, I don't know why, I mean, I used to when I was younger, but nothing seems to, perhaps I've seen too much and done too much, I don't know, I never sit and cry over anything. (Woman)

She attributed her emotional coping efforts to the fact that she is "not very emotional", a quality described in irreversible terms.

Other participants identified their professional lives as being particularly formative for their personality, and therefore their coping strategies. Several men spoke of their time in the

For peer review only - http://bmjopen.bmj.com/site/about/guidelines.xhtml

military, linking the job to their mindset, behaviours, and physicality: "I was in the military police for years and being in the military, it's a life…I was getting on with things, you know you just make do and make the best of the situation. (Man). This man illustrated "getting on with things" by describing the sit-ups and press-ups he did.

The role of personality was also emphasised by those describing their struggles to manage after stroke. They spoke of their personal traits and capabilities as stable from before to after their stroke, but now in conflict with the conditions of their life. One survivor said during her interview that being dependent was "against my nature" and that the occurrences of reliance on others provoked sadness as it reminded her of "everything that happened". She spoke about the challenges she faced in maintaining previous roles:

> Just very frustrated, helpless really, I think frustration because I'm not the kind, I've never been the kind of person to ask for help, you know, I'd always just got on and done things, you know and I was having to rely on, you know, my husband to, I'm looking at him and saying, and he's having to put the words there for me and I'm saying "Yeah, that's what I meant", you know and then I think about a week afterwards I said to my son "Oh would you like, would you like an omelette for tea?" And he said "Yeah, but I can do that mum", I said "No, I'm going to do you an omelette", and I went in the kitchen and he said "What's the matter?" And I went "[tuts] I don't know how to do it". (Woman)

The repetition of the dialogue with her son about making an omelette signifies the salient gap between her perceived personal qualities and her capabilities after stroke, a gap that runs

For peer review only - http://bmjopen.bmj.com/site/about/guidelines.xhtml

contrary to her "nature" to be independent, active and contributing to the care of others rather than receiving care herself. Appeal to fixed personal qualities served in this way as a narrative resource for explaining the meaning of present challenges, through reference to life before the stroke. The continuity of personality provided a way of both displaying and appraising discontinuities.

Discussion

Compared to research on coping with current threats, there has been much less attention to preventative coping. We sought to examine how stroke survivors characterise their coping process in the context of secondary prevention. In our data, themes of personal responsibility and self-sufficiency featured strongly. So did the figure of the doctor as adjudicator of being a 'good' stroke survivor. When compared to the other challenges faced by our participants, secondary prevention appeared particularly 'medicalized': both the criteria for complying and the judgement of success were characterised as external to participants, though the extent of success was held to be a fundamentally individual matter.

In this regard, we were struck that no participant in this study mentioned advice or conversations with healthcare professions regarding the possible emotional effects of being at

For peer review only - http://bmjopen.bmj.com/site/about/guidelines.xhtml

increased risk of a future stroke. Doctors loomed large in the imaginations of our participants, but are not sensed to be especially accessible. Other healthcare professionals were mentioned at times, but not characterised as relevant to preventative coping efforts. This finding regarding secondary prevention aligns with previous studies have identified that stroke survivors living in the community often report they have little support from health care providers for recovery.[4,29]. It is interesting to place together with findings that suggest healthcare professionals perceive themselves as attentive and responsive to the emotional effects on patients of being at increased risk of a future stroke,[30] signalling a potential disconnect between the perceptions of patients and professionals.[31,32]

More generally, our findings align with the conclusions of other qualitative studies, which have highlighted constructions of self-responsibility and the doctor as adjudicator in the narratives of survivors about recovery. Our work extends these findings by showing that the themes continue to influence accounts of efforts to reduce subsequent cardiovascular risk. However we were also struck by some differences in how these themes resonated in our data compared to accounts, influenced by Foucault, in the existing literature. Disciplinary power was not operating, as for Foucault, to remake the individuals in the image proposed by medical knowledge as normal as opposed to abnormal. Rather, self-responsibility was frequently

For peer review only - http://bmjopen.bmj.com/site/about/guidelines.xhtml

asserted through emphasis on continuity, rather than on remaking the self. A primary means used by survivors to characterising their efforts to be ‘good’ was to highlight the strengths they could draw upon from their live before stroke. In his late work, it is notable that Foucault himself found his characterisation of discipline too monolithic, and advocated the need to attend closely to exactly how self-responsibility was being conceptualised. He argued that efforts to strive to be good might operate quite differently, depending on what is construed as the ‘ethical substance’: “the way in which the individual has to constitute this or that part of himself as the prime material of his moral conduct”.[33] In our data, the prime material of the survivor was not constituted as requiring change in order to pursue preventative coping: rather, the resources made available by a stable personality were characterised as needing to be turned towards these challenges.

This narrative emphasis on continuity of the self stood in contradiction to the numerous implicit ways that speakers acknowledged major disruptions in their lives and self-concept. This is consistent with previous findings on how survivors talk about life after stroke. Previous studies have documented that stroke survivors’ sense of biographical continuity is shaped by the extent to which the stroke has impacted their self-image.[34,35,36] Our findings suggest a bidirectional relationship, in which self-image may be preserved through an insistence on continuity, and the sequestration of recognition of change.

For peer review only - http://bmjopen.bmj.com/site/about/guidelines.xhtml

Participants characterised their personality traits as something they drew on as individuals to help them in preventative coping. The descriptions were of these traits as being external to and unaffected by their stroke, even if this trait cannot be expressed in the same way as before the stroke. Such accounts may reflect cultural understandings, put forward in twentieth century psychology and circulated into popular discourse, in which traits are essential and independent of what they encounter.[37] Some of those that described struggling in their coping efforts, as in previous research,[3] contrasted their conception of the former efficacy of their own personal traits and abilities with what they struggled with now. The disruption for them appears to be the inability to behave in ways congruent with their personality. It seems plausible that if stroke survivors consider personal traits as constants in their lives, those that then describe their inability to function or participate as they had previously may consider themselves at fault in some way for their own condition. Furthermore, behaviour-change interventions or forms of therapy such as acceptance and commitment approaches could seem futile to participants if these changes would conflict with their notion of 'personality'. Our findings regarding the importance of narrative appeal to an essentialised notion of personality contribute to the growing literature exploring potential barriers and facilitators to changes in self-concept among stroke survivors.[38]

For peer review only - http://bmjopen.bmj.com/site/about/guidelines.xhtml

Limitations

There are several limitations to our study relevant to the interpretation of our results. Firstly, our interviews were with individual survivors. This may have foregrounded the theme of individual responsibility in our work, as in other qualitative studies. It may be anticipated that had dyadic interviews been undertaken, whether the survivor + family member or survivor + healthcare professional, the results would have foregrounded joint coping efforts towards secondary prevention.[39] Likewise, in our study and in other qualitative studies, the theme of individual striving to be good with the doctor as adjudicator may also have been less prominent or figured quite differently if dyadic interviews had been undertaken. For instance, the role of family members as additional adjudicators may have been more prominent in a joint interview between survivor and spouse. Our findings therefore relate primarily to the individual's account of their preventative coping efforts, and there may be important differences to the assumptions that guide joint coping activities with others, most notably family members.

This raises the wider limitation that our narrative data is from a single time point and lacking triangulation with observational findings. So for instance, our reflections on the potential for a static image of personality to hinder utilisation of behaviour-change interventions or update their self-concept in adaptive ways therefore remain speculative. There may also be relevant limitations in the transferability of our findings across healthcare settings,

For peer review only - http://bmjopen.bmj.com/site/about/guidelines.xhtml

including culturally specific aspects of our participants' accounts. Whilst accounts by stroke survivors of the need to strive to be good, with doctors as adjudicators, have been reported across various countries, our study is the first to highlight the theme of personality traits in survivors accounts of preventative coping. Pickersgill has proposed that psychological discourses of personality have become prominent in the UK.[37] So we do not know the extent to which this finding may have been shaped by the national context, or whether essentialised notions of self, if not the idea of personality, are relevant more widely to how survivors think about preventative coping.

Conclusions

Our study reported from interviews with stroke survivors, conducting a thematic analysis to explore preventative coping. Two themes were generated from analysis of the transcripts. A first theme was of the individual's efforts to strive to be 'good', as adjudicated by doctors. Coping was figured as an individual's task, with little reference to joint efforts with family members or healthcare professionals. Indeed, doctors loomed large in the imaginations of our participants, but were not sensed to be especially accessible. Family members and other healthcare professionals, whilst discussed in other contexts, were not generally characterised

For peer review only - http://bmjopen.bmj.com/site/about/guidelines.xhtml

as contributing to preventative coping efforts. The theme of individualised efforts to be 'good' aligns with other interview studies of survivors' rehabilitation, extending this observation to the context of secondary prevention. A second, novel theme was of reference by participants to 'personality' as a source of felt continuity and a resource for preventative coping. Appeals to essentialised notions of self may help survivors sustain feelings of biographical coherence, but may also prove an obstacle to behaviour change, as well as adaptive alterations in self-concept.

Contributorship statement: The study was conceived and designed by PSR, EN, FC, RM, JM and RD. PSR, FC, RM, JM and RD are academic researchers; EN was a student intern. Transcripts were coded by PSR and EN. PSR, RD and JM drafted the manuscript. All authors have read and approved the final manuscript.

Competing interests: None

Funding: This study is funded by the National Institute for Health Research's (NIHR) Programme Grant for Applied Research titled 'Developing primary care services for stroke survivors' (reference PTC-RP-PG-0213–20001). The views expressed are those of the authors and not necessarily those of the NIHR or the Department of Health and Social Care.

For peer review only - http://bmjopen.bmj.com/site/about/guidelines.xhtml

JM is an NIHR Senior Investigator.

Data sharing statement: Data are available upon reasonable request. Contact Jonathan Mant, jm677@medschl.cam.ac.uk

Ethics statement: Favourable ethical opinion for the research was gained on 19 December 2017 from Yorkshire and the Humber-Bradford Leeds NHS Research Ethics Committee. Approval to start was given by the Health Research Authority (HRA) on 21 December 2017, prior to the recruitment of participants commencing at any NHS site. Patient recruitment started in March 2018. IRAS project ID: 233891. Protocol number: RG71908. REC reference: 17/YH/0441.

References

1. Bhatnagar P, Wickramasinghe K, Wilkins E, Townsend N. Trends in the epidemiology of cardiovascular disease in the UK. Heart. 2016;102(24):1945-52.. https://doi.org/10.1136/heartjnl-2016-309573
2. Clarke P, Black SE. Quality of life following stroke: negotiating disability, identity, and resources. Journal of Applied Gerontology. 2005;24(4):319-36. https://doi.org/10.1177/0733464805277976

For peer review only - http://bmjopen.bmj.com/site/about/guidelines.xhtml

3. Crowfoot G, van der Riet P, Maguire J. Real-life experiences of people with transient ischaemic attack or minor stroke: A qualitative literature review. Journal of clinical nursing. 2018;27(7-8):1381-98. https://doi.org/10.1111/jocn.14271

4. Boger EJ, Demain SH, Latter SM. Stroke self-management: A focus group study to identify the factors influencing self-management following stroke. International Journal of Nursing Studies. 2015;52(1):175-87. https://doi.org/10.1016/j.ijnurstu.2014.05.006

5. Ellis J, Boger E, Latter S, Kennedy A, Jones F, Foster C, Demain S. Conceptualisation of the 'good'self-manager: A qualitative investigation of stakeholder views on the self-management of long-term health conditions. Social Science & Medicine. 2017;176:25-33. https://doi.org/10.1016/j.socscimed.2017.01.018

6. Fletcher S, Kulnik ST, Demain S, Jones F. The problem with self-management: Problematising self-management and power using a Foucauldian lens in the context of stroke care and rehabilitation. PloS one. 2019 Jun 19;14(6):e0218517. https://doi.org/10.1371/journal.pone.0218517

For peer review only

For peer review only - http://bmjopen.bmj.com/site/about/guidelines.xhtml

7. Vassilev I, Rogers A, Todorova E, Kennedy A, Roukova P. The articulation of neoliberalism: narratives of experience of chronic illness management in Bulgaria and the UK. Sociology of Health & Illness. 2017;39(3):349-64. https://doi.org/10.1111/1467-9566.12488

8. Abrahamson V, Wilson P. Positioning the six-month review in the recovery process post-stroke: The ideology of personal responsibility. Health & social care in the community. 2019;27(1):249-59. https://doi.org/10.1111/hsc.12677

9. Lazarus, R., Folkman, S. Stress, Appraisal and Coping. New York: Springer, 1984, p.34

10. Kuo BC. Collectivism and coping: Current theories, evidence, and measurements of collective coping. International Journal of Psychology. 2013;48(3):374-88. https://doi.org/10.1080/00207594.2011.640681

11. Vassilev I, Rogers A, Blickem C, Brooks H, Kapadia D, Kennedy A, Sanders C, Kirk S, Reeves D. Social networks, the 'work' and work force of chronic illness self-management: a survey analysis of personal communities. PloS one. 2013;8(4):e59723. https://doi.org/10.1371/journal.pone.0059723

For peer review only - http://bmjopen.bmj.com/site/about/guidelines.xhtml

12. Faircloth CA, Boylstein C, Rittman M, Young ME, Gubrium J. Sudden illness and biographical flow in narratives of stroke recovery. Sociology of health & illness. 2004;26(2):242-61. https://doi.org/10.1111/j.1467-9566.2004.00388.x

13. Simeone S, Savini S, Cohen MZ, Alvaro R, Vellone E. The experience of stroke survivors three months after being discharged home: A phenomenological investigation. European Journal of Cardiovascular Nursing. 2015;14(2):162-9. https://doi.org/10.1177/1474515114522886

14. Rochette A, Tribble DS, Desrosiers J, Bravo G, Bourget A. Adaptation and coping following a first stroke: a qualitative analysis of a phenomenological orientation. International Journal of Rehabilitation Research. 2006;29(3):247-9. https://doi.org/10.1097/01.mrr.0000230051.24228.c8

15. Finch E, Foster M, Fleming J. Disrupted biographies: making sense of minor stroke after hospital discharge. Disability and Rehabilitation. 2020:1-8. https://doi.org/10.1080/09638288.2019.1708980

16. Carlsson GE, Möller A, Blomstrand C. Managing an everyday life of uncertainty–a qualitative study of coping in persons with mild stroke. Disability and Rehabilitation. 2009;31(10):773-82. https://doi.org/10.1080/09638280802638857

For peer review only - http://bmjopen.bmj.com/site/about/guidelines.xhtml

17. Dalvandi A, Heikkilä K, Maddah SS, Khankeh HR, Ekman SL. Life experiences after stroke among Iranian stroke survivors. International nursing review. 2010;57(2):247-53. https://doi.org/10.1111/j.1466-7657.2009.00786.x

18. Pallesen H. Body, coping and self-identity. A qualitative 5-year follow-up study of stroke. Disability and Rehabilitation. 2014;36(3):232-41. https://doi.org/10.3109/09638288.2013.788217

19. Persson HC, Törnbom K, Sunnerhagen KS, Törnbom M. Consequences and coping strategies six years after a subarachnoid hemorrhage–A qualitative study. PLoS One. 2017;12(8):e0181006. https://doi.org/10.1371/journal.pone.0181006

20. Jamison J, Graffy J, Mullis R, Mant J, Sutton S. Barriers to medication adherence for the secondary prevention of stroke: a qualitative interview study in primary care. British journal of general practice. 2016;66(649):e568-76. https://doi.org/10.3399/bjgp16X685609

21. Ravenhill G, Gkanasouli L, Lawrence M. Experiences of transient ischaemic attack diagnosis and secondary prevention: a qualitative review. British Journal of Neuroscience Nursing. 2019;15(5):S14-25. https://doi.org/10.12968/bjnn.2019.15.Sup5.S14

For peer review only

For peer review only - http://bmjopen.bmj.com/site/about/guidelines.xhtml

22. Parappilly BP, Mortenson WB, Field TS, Eng JJ. Exploring perceptions of stroke survivors and caregivers about secondary prevention: a longitudinal qualitative study. Disability and Rehabilitation. 2020;42(14):2020-6. https://doi.org/10.1080/09638288.2018.1544296

23. Schwarzer R, Knoll N. Positive coping: Mastering demands and searching for meaning. In S. J. Lopez & C. R. Snyder (Eds.), *Positive psychological assessment: A handbook of models and measures.* (pp. 393–409). American Psychological Association. 2003. https://doi.org/10.1037/10612-025

24. Intercollegiate Stroke Working Party. National Clinical Guidelines for Stroke, 5th Edition. Royal College of Physicians. 2016

25. Braun V, Clarke V. Using thematic analysis in psychology. Qualitative Research in Psychology. 2006;3(2):77-101. https://doi.org/10.1191/1478088706qp063oa

26. Frith H, Gleeson K. Clothing and embodiment: Men managing body image and appearance. Psychology of Men & Masculinity. 2004;5(1):40. https://doi.org/10.1037/1524-9220.5.1.40

For peer review only

27. MacQueen, K. M., McLellan, E., Kay, K., & Milstein, B.. Codebook development for team-based qualitative analysis. *CAM Journal*, 1998;10(2):31–36. https://doi.org/10.1177/1525822X980100020301

28. Saldaña, J. (2015). *The coding manual for qualitative researchers*. London: Sage.

29. Croot EJ, Ryan TW, Read J, Campbell F, O'Cathain A, Venables G. Transient ischaemic attack: a qualitative study of the long term consequences for patients. BMC Fam Pract. 2014;15:174. https://doi.org/10.1186/s12875-014-0174-9

30. Morris R. Meeting the psychological needs of community-living stroke patients and carers: a study of third sector provision. Disability and Rehabilitation. 2016;38(1):52-61. https://doi.org/10.3109/09638288.2015.1014932

31. Playford ED, Siegert R, Levack W, Freeman J. Areas of consensus and controversy about goal setting in rehabilitation: a conference report. Clinical Rehabilitation. 2009;23(4):334-44. https://doi.org/10.1177/0269215509103506

32. Kidd L, Booth J, Lawrence M, Rowat A. Implementing supported self-management in community-based stroke care: a secondary analysis of nurses' perspectives. Journal of Clinical Medicine. 2020;9(4):985. https://doi.org/10.3390/jcm9040985

For peer review only

33. Foucault, M. (1985) The Use of Pleasure, trans. Robert Hurley, London: Penguin, p.26

34. Pound P, Gompertz P, Ebrahim S. Illness in the context of older age: the case of stroke. Sociology of health & illness. 1998;20(4):489-506. https://doi.org/10.1111/1467-9566.00112

35. Large R, Samuel V, Morris R. A changed reality: Experience of an acceptance and commitment therapy (ACT) group after stroke. Neuropsychological rehabilitation. 2019;30(8):1477-1496. https://doi.org/10.1080/09602011.2019.1589531

36. Hutton L, Ownsworth T. A qualitative investigation of sense of self and continuity in younger adults with stroke. Neuropsychological Rehabilitation. 2019;29(2):273-88. https://doi.org/10.1080/09602011.2017.1292922

37. Pickersgill M. How personality became treatable: The mutual constitution of clinical knowledge and mental health law. Social Studies of Science. 2013;43(1):30-53. https://doi.org/10.1177/0306312712457722

38. Higgs R, Soundy A. Factors which influence risk taking and the evolution of social-identity in stroke narratives: a thematic synthesis. Behavioral Sciences. 2020;10(2):46. https://doi.org/10.3390/bs10020046

For peer review only - http://bmjopen.bmj.com/site/about/guidelines.xhtml

39. Klevan T, Jonassen R, Sælør KT, Borg M. Using dyadic interviews to explore recovery as collaborative practices: challenging the epistemic norm of the single person perspective. International Journal of Qualitative Methods. 2020;19. https://doi.org/10.1177/1609406920967868

For peer review only

For peer review only - http://bmjopen.bmj.com/site/about/guidelines.xhtml

Standards for Reporting Qualitative Research

1. Title x
2. Abstract x
3. Problem formulation x
4. Research question x
5. Qualitative approach x
6. Researcher characteristics x
7. Context x
8. Sampling x
9. Ethical issues x
10. Data collection methods x
11. Data collection instruments x
12. Units of study x
13. Data processing x
14. Data analysis x
15. Trustworthiness x
16. Synthesis x
17. Empirical data x
18. Integration with prior work x
19. Limitations x
20. Conflicts of interest x
21. Funding x

For peer review only

BMJ Open

Accounts of preventative coping: An interview study of stroke survivors on General Practice registers

Journal:	*BMJ Open*
Manuscript ID	bmjopen-2021-058441.R1
Article Type:	Original research
Date Submitted by the Author:	20-May-2022
Complete List of Authors:	Scott Reid, Peter; University of Cambridge Primary Care Unit<br>Neville, Emma; University of Cambridge Primary Care Unit<br>Cater, Frances; University of Cambridge Primary Care Unit<br>Mullis, Ricky; University of Cambridge, Department of Public Health and Primary Care<br>Mant, Jonathan; University of Cambridge, General Practice and Primary Care Research Unit<br>Duschinsky, Robbie; University of Cambridge Primary Care Unit
**Primary Subject Heading**:	Cardiovascular medicine
Secondary Subject Heading:	Rehabilitation medicine
Keywords:	Stroke < NEUROLOGY, CARDIOLOGY, REHABILITATION MEDICINE

SCHOLARONE™
Manuscripts

I, the Submitting Author has the right to grant and does grant on behalf of all authors of the Work (as defined in the below author licence), an exclusive licence and/or a non-exclusive licence for contributions from authors who are: i) UK Crown employees; ii) where BMJ has agreed a CC-BY licence shall apply, and/or iii) in accordance with the terms applicable for US Federal Government officers or employees acting as part of their official duties; on a worldwide, perpetual, irrevocable, royalty-free basis to BMJ Publishing Group Ltd ("BMJ") its licensees and where the relevant Journal is co-owned by BMJ to the co-owners of the Journal, to publish the Work in this journal and any other BMJ products and to exploit all rights, as set out in our licence.

The Submitting Author accepts and understands that any supply made under these terms is made by BMJ to the Submitting Author unless you are acting as an employee on behalf of your employer or a postgraduate student of an affiliated institution which is paying any applicable article publishing charge ("APC") for Open Access articles. Where the Submitting Author wishes to make the Work available on an Open Access basis (and intends to pay the relevant APC), the terms of reuse of such Open Access shall be governed by a Creative Commons licence – details of these licences and which Creative Commons licence will apply to this Work are set out in our licence referred to above.

Other than as permitted in any relevant BMJ Author's Self Archiving Policies, I confirm this Work has not been accepted for publication elsewhere, is not being considered for publication elsewhere and does not duplicate material already published. I confirm all authors consent to publication of this Work and authorise the granting of this licence.

For peer review only

Accounts of preventative coping: An interview study of stroke survivors on General Practice registers

For peer review only

Author and Co-author's name: Peter Scott Reid, Emma Neville, Frances Cater, Ricky Mullis, Jonathan Mant & Robbie Duschinsky

Corresponding author: Robbie Duschinsky, East Forvie Site, University of Cambridge, Cambridge, CB2 0PY, 01223 746426
rd522@medschl.cam.ac.uk

Affiliation all authors: Primary Care Unit, University of Cambridge, East Forvie Site, Cambridge, CB2 0PY

Key words:
Chronic illness, coping, secondary prevention, self-concept, stroke, thematic analysis

Word count: 4089 (main text: excluding abstract; table; references and statements at end of paper)

For peer review only - http://bmjopen.bmj.com/site/about/guidelines.xhtml

Abstract

Objectives: Preventative coping is an underexplored aspect of coping behaviour. Specifically, coping is a key concern in stroke survivor accounts, but this has yet to be investigated with reference to secondary prevention.

Design: Secondary analysis of a qualitative dataset comprising semi-structured interviews of 22 stroke survivors recruited from five General Practices in the East of England. The topic guide included exploration of advice and support given by their doctor on medication and lifestyle. The interviews were coded using thematic analysis.

Results: The accounts emphasised individual responsibility. Two key themes were identified, which foregrounded the role of self-concept for coping: a) striving to be ‘good’, b) appeal to ideas of 'personality'. In the former, preventative behaviour was depicted in moralistic terms,

For peer review only

with the doctor as an adjudicator. In the latter, participants attributed their coping behaviour to their personality, which might help or hinder these efforts.

Conclusions: We highlight that coping was characterised by survivors as something enacted by the individual self, and consider how constructions of self may impact preventative coping efforts.

Strengths and limitations

- The study drew upon detailed qualitative characterisation of the perceptions of stroke survivors of aspects of coping with reference to secondary prevention
- The analytic approach foregrounded both lines of consistency and variation in how stroke survivors spoke about their experiences
- The data relate to participants' perceptions of their coping, rather than their coping practices
- From a single interview with each participants, we cannot draw firm conclusions about how coping is embedded in aspects of participants lives over time.

Competing interests: N/A

For peer review only - http://bmjopen.bmj.com/site/about/guidelines.xhtml

For peer review only

Key words:
Chronic illness, coping, secondary prevention, self-concept, stroke, thematic analysis

For peer review only

For peer review only - http://bmjopen.bmj.com/site/about/guidelines.xhtml

Introduction

Mortality from stroke in the UK is declining but the prevalence of stroke is rising due to improved survival rates and an aging population.[1] Stroke frequently results in major life changes and can influence a person physically, psychologically and socially.[2,3] Stroke survivors living in the community often report they have little support – but also anticipate little support since they feel they are obliged to take personal responsibility for managing their own recovery.[4,5] For instance, Fletcher et al. found themes of self-responsibility and the power of medical authority in their study of stroke survivors.[6] They interpreted their findings in terms of Foucauldian ideas of power, in which survivors were enjoined to discipline themselves in order to effect a successful recovery, and relieve pressure on health and social care services. Commentators have suggested that this construction of stroke survivorship reflects broader discourses on chronic illness that highlight individual responsibility and self-sufficiency, under guidance from distant experts but with limited service provision.[7,8]

Understanding stroke survivors' perspectives through the lens of 'coping' can add knowledge of the needs of this patient group as well as inform services for stroke survivors. Perhaps the classic definition of coping was offered in the 1980s by Lazarus and Folkman, who characterise it as the "thoughts and behaviours that people use to manage the internal and external demands of situations that are appraised as stressful".[9] 'Coping' used in this way is

For peer review only - http://bmjopen.bmj.com/site/about/guidelines.xhtml

the attempt to manage challenges in life, rather than necessarily being successful in that attempt. Most work on coping has addressed the challenge of how individuals manage current problems. Yet in recent years scholarship has increasingly emphasised coping as frequently a joint undertaking.[10,11] Sometimes this is explicit, with actors aware of the coordinated or collective work 'we' do to cope. Sometimes this is implicit, with actors less able to willingly acknowledge the contribution of others to 'my' coping with the demands of situations. Such accounts have been frequently found to be shaped by perceptions of the source of the stressful demands, and on attributions about others, and the extent to which the demands are felt to be a disruption or in continuity with past experiences.[12]

Much qualitative research on stroke survivors has been focused on the immediate period up to one year following the stroke, thus prioritising the initial stages of disruption.[13,14,15,16,17] A few studies have conducted later follow-up, but with a focus on how survivors deal with the effects of the initial stroke and not on secondary prevention.[18,19] More recently, secondary prevention following stroke has seen attention by a small number of studies and reviews,[20,21,22] but these acknowledge that the topic remains underexplored. In particular, there has been relatively little attention to 'preventative coping', which can be defined as the

For peer review only - http://bmjopen.bmj.com/site/about/guidelines.xhtml

efforts individuals make to prepare for uncertain events in the long run by building up resources that will result in less strain in the future by minimising the event's severity.[23]

Guidelines emphasise the importance of changes to lifestyle and the role of medication in reducing the risk of having a second stroke.[24] While they stress the importance of a personalised approach, this is in terms of what to recommend rather than how to optimise chances that the recommendations will be adopted. Analysis of reasons for this sub-optimal use that explore patient perspectives have tended to focus on aspects such as self-care ability, knowledge and understanding, rather than the role of preventative coping strategies. Furthermore, what attention is given to preventative coping tends to treat it as the action of individuals, rather than a joint or jointly-coordinated activity with others. Better understanding of preventative coping strategies might lay the ground for more nuanced ways of developing approaches to supporting secondary prevention after stroke. It was recognised that a qualitative dataset of interviews of stroke survivors on general practice stroke registers that had been performed to inform a primary care based model of longer term care for stroke might provide insights into these issues. This article therefore sets out to answer the question: how do stroke survivors characterise their coping process in the context of secondary prevention?

For peer review only - http://bmjopen.bmj.com/site/about/guidelines.xhtml

Methods

Study design and participants

For peer review only

For peer review only - http://bmjopen.bmj.com/site/about/guidelines.xhtml

A secondary analysis was conducted on transcripts of semi-structured interviews carried out to inform the design of an intervention aimed at improving primary care after stroke. The aim of the original study was to understand how primary care meets stroke survivors' needs, and how it could better address them in the future. Participants were recruited from five General Practices in the East of England. Our target was to recruit 20 stroke survivors. The participating General Practices generated a list of people on their stroke register, and a random sample of these were reviewed by the local general practitioner to confirm eligibility. Inclusion criteria were: 1) Confirmed diagnosis of stroke from survivor's records. 2) Has a good understanding of English. 3) Has the capacity to provide written informed consent. Exclusion criteria were: 1) Survivors considered by their GP to be sufficiently ill that it would make participation difficult. Expressive aphasia was not a basis for exclusion. Twenty five prospective participants were sent a study invitation pack by each of the five practice administrators, with replies being sent to the study team. The invitation packs included a covering letter, a participant information sheet, a reply slip, and a short questionnaire requesting demographic information, and details of their stroke (how long ago; level of disability). Respondents who indicated they were willing to take part were then selected using maximum variety sampling (at least 3 patients from each practice; at least 2 from each

For peer review only - http://bmjopen.bmj.com/site/about/guidelines.xhtml

tertile of Index of Multiple Deprivation score of the East of England; at least 8 of each sex; at least 2 participants from each age range: < 65 years; 65-79 years; 80 years or over; at least 2 participants who had a stroke within the previous year and 2 who had their stroke over a year previously. 22 participants were interviewed (Table 1).

For peer review only

Data collection

Participants who indicated they were willing to take part who were selected for inclusion were contacted by telephone by a researcher to answer any questions they might have. If the participants were still willing to proceed, then a suitable interview time was arranged. Before the interview started, the researcher confirmed that responses would be anonymous and checked what might be done to make the interview as easy as possible for the participant (e.g. presence of a carer; where the interviewer sat). A consent form was signed. Interviews took place February-May 2016.

Table 1 – Participant information

**Total subjects**	22
Men	11
Women	11
**Age**	

Range	49 to 93
Mean	73
Median	70
Standard Deviation	11.8
**Barthel Index as a measure of disability (Total score, out of 100)**	
Range	50 to 100
Mean	89.8
Median	100.0
**Length of time since stroke**	
Range	3 months to 22 years 6 months
Mean	7 years
Median	3 years
Standard Deviation	5 years 11 months

Interviews were conducted in participants' homes. Interviews were performed by an experienced qualitative researcher who was not a health care professional and was not known to any of the participants. An interview guide was used with questions focused on life after stroke. This included questions on family and social relations, the participants' sense of self, their emotions, interactions with health care professionals, and how they reflected on their experiences. Questions addressed both the nature of small practical adjustments required and participants' wider sense of their experiences. Follow-up questions were used to probe for relevant details.

For peer review only - http://bmjopen.bmj.com/site/about/guidelines.xhtml

Public and Patient Involvement

A PPI member was involved in developing the research as a co-applicant. The results of the interviews were fed back to a multi-disciplinary intervention development group that included PPI representation who helped placed the results in context. The research was overseen by an independent steering committee that included a PPI member. The open ended questions used in the interviews were informed by meetings with community stroke survivor and carer support groups.

Thematic analysis

All interviews were recorded and transcribed verbatim by professional transcribers prior to analysis. All transcripts obtained for the original study were included in this secondary analyis. Thematic Analysis (TA) is a "technique for identifying, analysing and reporting patterns (themes) within data".[25] Researchers using TA have praised its capacity to tease out contradictions between statements saying one thing and statements suggesting another, and to explore how people construct and give account of their attitudes.[26] TA was combined with a constant comparative approach, using existing theory and literature from coping and qualitative work on life after stroke to sensitise our development of codes, theme generation, and analysis.

For peer review only - http://bmjopen.bmj.com/site/about/guidelines.xhtml

Two co-authors collaborated on the development of the codebook for the TA, which contained the code, a definition, guidelines for when and when not to use the code, and examples.[27] The first author then coded all of the verbatim interviews using NVivo software. Themes were identified as aggregates of codes, speaking to broader ideas in the literature. These themes were revised and refined several times by looking at how they held up as valid interpretations of the data when referring back to the transcripts, and on the basis of discussion with the rest of the study team.[28]

Findings

Participants all spoke about their preventative coping efforts. Some employed the word 'coping' itself while others described how they evaluated and dealt with the challenges of secondary prevention using different terms. What was most striking about the accounts was the strong emphasis of participants on individual efforts. There was little explicit acknowledgement that coping might be a jointly pursued activity, though this was implicit. Rather, the two most prominent themes with respect to secondary prevention were a) the need for the self to strive to be 'good', and b) the role of 'personality' as a resource for these efforts.

For peer review only - http://bmjopen.bmj.com/site/about/guidelines.xhtml

Striving to be ‘good’

Across the interviews, participants employed the language of discipline: ‘don’t eat silly things’, ‘strict’, ‘moderation’, ‘it’s me own fault’, ‘control’, ‘careful’. This underwrote their depictions of preventative behaviour in moralistic terms, and the weight of expectation they felt to be ‘good’. Participants often discussed how doctors had evaluated their efforts to change their health-affecting behaviours and habits such as eating, drinking, and exercise. One interviewee relayed the advice given to her about losing weight while coupling it with the doctor’s approval of her habits and behaviours:

> They did say to me “Try and lose a little bit of weight”, but you know I mean he came, the consultant came in on the day I came home and he said “This is where I should be saying to you stop smoking, stop drinking, there’s your cholesterol tablets and there’s your blood pressure tablets” and he said “I can’t” because I don’t smoke, I’ve never smoked, the occasional drink and the other two were fine so he said “You know, try and get a little bit of weight off and just have your exercise,” and yeah. (Woman, aged 45-54)

The participant here depicts their successful prevention efforts, constructed in contrast to the hypothetical and less adherent patient that her doctor described. Another patient described interactions with his doctor about changing his drinking behaviour to better prevent a stroke:

> I wasn’t happy with the doctor I saw, she kept telling me oh you can have another stroke very quickly, you know. Yeah you’ll soon have another stroke if you don’t do this and you don’t do that. If you do too much you’ll have another stroke, you know that don’t you. If you do…and this was continual, this woman kept telling me this. I do drink more than I should do, I admit that, not a vast amount, but I do drink more than I should do. I drink

For peer review only

For peer review only - http://bmjopen.bmj.com/site/about/guidelines.xhtml

wine, and I do enjoy a nice drink. But she said oh, if you have…drink, you know, more than one glass full you're going to have a stroke. (Man, aged 55-64)

The two accounts above, as with most of our interviews, depicted doctors as external adjudicators of individuals' coping efforts. Though one (woman, aged 45-54) speaks of broadly being affirmed and the other (man, aged 55-64) of feeling chastised for a perceived lack of effort, they both draw on doctors as arbitrating the validity of their attempts to prevent a future stroke and how 'good' they have been. In contrast to the prominent role given to doctors, other healthcare professionals and family members were mentioned in other parts of the interview but not generally in discussions of secondary prevention.

One curious aspect of the accounts was that, despite commonalities in the language of discipline used, survivors did not necessarily give the same description of what being 'good' or 'bad' entailed. Participants were variously concerned with striving to be good in their medication, smoking, weight, exercise, or alcohol use – or some combination of the above, though very rarely with more than a few. What the accounts had in common was the conception that participants felt that they should be *striving* for these changes or behaviours – even if they currently fall short or disagree with their doctor's judgement of success. This theme suggests that participants construct and use categories of 'good' and 'bad' behaviour in relation to

For peer review only - http://bmjopen.bmj.com/site/about/guidelines.xhtml

prevention, and that moralistic terms are even used by those survivors feeling they have behaved in ways that are not ‘good’.

Appeal to personality

A consistent theme throughout the interviews was survivors’ appeal to the idea of ‘personality’ or more generally stable traits and qualities in accounting for their coping efforts. Participants consistently described the self as fixed rather than a malleable set of traits. They perceived this stability as of fundamental importance in hindering or helping their preventative coping attempts, and in explaining how their experiences prior to stroke shaped the way they dealt with the challenges they faced. One of the women in the sample was asked how she had coped emotionally since the stroke:

> I'm not a very emotional person, I don't think, really. I mean, I just think things in my head, but I never cry, never, ever cry. I don't know why, I think that's something, I don't know why, I mean, I used to when I was younger, but nothing seems to, perhaps I've seen too much and done too much, I don't know, I never sit and cry over anything. (Woman, aged 75-84)

She attributed her emotional coping efforts to the fact that she is “not very emotional”, a quality described in irreversible terms.

Other participants identified their professional lives as being particularly formative for their personality, and therefore their coping strategies. Several men spoke of their time in the

For peer review only - http://bmjopen.bmj.com/site/about/guidelines.xhtml

military, linking the job to their mindset, behaviours, and physicality: “I was in the military police for years and being in the military, it’s a life…I was getting on with things, you know you just make do and make the best of the situation. (Man, aged 55-64). This man illustrated “getting on with things” by describing the sit-ups and press-ups he did.

The role of personality was also emphasised by those describing their struggles to manage after stroke. They spoke of their personal traits and capabilities as stable from before to after their stroke, but now in conflict with the conditions of their life. One survivor said during her interview that being dependent was “against my nature” and that the occurrences of reliance on others provoked sadness as it reminded her of “everything that happened”. She spoke about the challenges she faced in maintaining previous roles:

> Just very frustrated, helpless really, I think frustration because I’m not the kind, I’ve never been the kind of person to ask for help, you know, I’d always just got on and done things, you know and I was having to rely on, you know, my husband to, I’m looking at him and saying, and he’s having to put the words there for me and I’m saying “Yeah, that’s what I meant”, you know and then I think about a week afterwards I said to my son “Oh would you like, would you like an omelette for tea?” And he said “Yeah, but I can do that mum”, I said “No, I’m going to do you an omelette”, and I went in the kitchen and he said “What’s the matter?” And I went “[tuts] I don’t know how to do it”. (Woman, aged 45-54)

The repetition of the dialogue with her son about making an omelette signifies the salient gap between her perceived personal qualities and her capabilities after stroke, a gap that runs

For peer review only

contrary to her "nature" to be independent, active and contributing to the care of others rather than receiving care herself. Appeal to fixed personal qualities served in this way as a narrative resource for explaining the meaning of present challenges, through reference to life before the stroke. The continuity of personality provided a way of both displaying and appraising discontinuities.

Discussion

Compared to research on coping with current threats, there has been much less attention to preventative coping. We sought to examine how stroke survivors characterise their coping process in the context of secondary prevention. In our data, themes of personal responsibility and self-sufficiency featured strongly. So did the figure of the doctor as adjudicator of being a 'good' stroke survivor. When compared to the other challenges faced by our participants, secondary prevention appeared particularly 'medicalized': both the criteria for complying and the judgement of success were characterised as external to participants, though the extent of success was held to be a fundamentally individual matter.

In this regard, we were struck that no participant in this study mentioned advice or conversations with healthcare professions regarding the possible emotional effects of being at

For peer review only - http://bmjopen.bmj.com/site/about/guidelines.xhtml

increased risk of a future stroke. Doctors loomed large in the imaginations of our participants, but are not sensed to be especially accessible. Other healthcare professionals were mentioned at times, but not characterised as relevant to preventative coping efforts. This finding regarding secondary prevention aligns with previous studies which have identified that stroke survivors living in the community often report they have little support from health care providers for recovery.[4,29]. It is interesting to place this together with findings that suggest healthcare professionals perceive themselves as attentive and responsive to the emotional effects on patients of being at increased risk of a future stroke,[30] signalling a potential disconnect between the perceptions of patients and professionals.[31,32]

More generally, our findings align with the conclusions of other qualitative studies, which have highlighted constructions of self-responsibility and the doctor as adjudicator in the narratives of survivors about recovery. Our work extends these findings by showing that the themes continue to influence accounts of efforts to reduce subsequent cardiovascular risk. However we were also struck by some differences in how these themes resonated in our data compared to accounts, influenced by Foucault, in the existing literature. Disciplinary power was not operating, as for Foucault, to remake the individuals in the image proposed by medical knowledge as normal as opposed to abnormal. Rather, self-responsibility was frequently

For peer review only - http://bmjopen.bmj.com/site/about/guidelines.xhtml

asserted through emphasis on continuity, rather than on remaking the self. A primary means used by survivors to characterising their efforts to be ‘good’ was to highlight the strengths they could draw upon from their life before stroke. In his late work, it is notable that Foucault himself found his characterisation of discipline too monolithic, and advocated the need to attend closely to exactly how self-responsibility was being conceptualised. He argued that efforts to strive to be good might operate quite differently, depending on what is construed as the ‘ethical substance’: “the way in which the individual has to constitute this or that part of himself as the prime material of his moral conduct”.[33] In our data, the prime material of the survivor was not constituted as requiring change in order to pursue preventative coping: rather, the resources made available by a stable personality were characterised as needing to be turned towards these challenges.

This narrative emphasis on continuity of the self stood in contradiction to the numerous implicit ways that speakers acknowledged major disruptions in their lives and self-concept. This is consistent with previous findings on how survivors talk about life after stroke. Previous studies have documented that stroke survivors’ sense of biographical continuity is shaped by the extent to which the stroke has impacted their self-image.[34,35,36] Our findings suggest a bidirectional relationship, in which self-image may be preserved through an insistence on continuity, and the sequestration of recognition of change.

For peer review only - http://bmjopen.bmj.com/site/about/guidelines.xhtml

Participants characterised their personality traits as something they drew on as individuals to help them in preventative coping. The descriptions were of these traits as being external to and unaffected by their stroke, even if this trait cannot be expressed in the same way as before the stroke. Such accounts may reflect cultural understandings, put forward in twentieth century psychology and circulated into popular discourse, in which traits are essential and independent of what they encounter.[37] Some of those that described struggling in their coping efforts, as in previous research,[3] contrasted their conception of the former efficacy of their own personal traits and abilities with what they struggled with now. The disruption for them appears to be the inability to behave in ways congruent with their personality. It seems plausible that if stroke survivors consider personal traits as constants in their lives, those that then describe their inability to function or participate as they had previously may consider themselves at fault in some way for their own condition. Furthermore, behaviour-change interventions or forms of therapy such as acceptance and commitment approaches could seem futile to participants if these changes would conflict with their notion of 'personality'. Our findings regarding the importance of narrative appeal to an essentialised notion of personality contribute to the growing literature exploring potential barriers and facilitators to changes in self-concept among stroke survivors.[38]

For peer review only - http://bmjopen.bmj.com/site/about/guidelines.xhtml

Limitations

There are several limitations to our study relevant to the interpretation of our results. Firstly, our interviews were with individual survivors. This may have foregrounded the theme of individual responsibility in our work, as in other qualitative studies. It may be anticipated that had dyadic interviews been undertaken, whether the survivor + family member or survivor + healthcare professional, the results would have foregrounded joint coping efforts towards secondary prevention.[39] Likewise, in our study and in other qualitative studies, the theme of individual striving to be good with the doctor as adjudicator may also have been less prominent or figured quite differently if dyadic interviews had been undertaken. For instance, the role of family members as additional adjudicators may have been more prominent in a joint interview between survivor and spouse. Our findings therefore relate primarily to the individual's account of their preventative coping efforts, and there may be important differences to the assumptions that guide joint coping activities with others, most notably family members.

This raises the wider limitation that our narrative data is from a single time point and lacking triangulation with observational findings. So for instance, our reflections on the potential for a static image of personality to hinder utilisation of behaviour-change interventions or update their self-concept in adaptive ways therefore remain speculative. There may also be relevant limitations in the transferability of our findings across healthcare settings,

For peer review only - http://bmjopen.bmj.com/site/about/guidelines.xhtml

including culturally specific aspects of our participants' accounts. Whilst accounts by stroke survivors of the need to strive to be good, with doctors as adjudicators, have been reported across various countries, our study is the first to highlight the theme of personality traits in survivors accounts of preventative coping. Pickersgill has proposed that psychological discourses of personality have become prominent in the UK.[37] So we do not know the extent to which this finding may have been shaped by the national context, or whether essentialised notions of self, if not the idea of personality, are relevant more widely to how survivors think about preventative coping.

Conclusions

Our study reported from interviews with stroke survivors, conducting a thematic analysis to explore preventative coping. Two themes were generated from analysis of the transcripts. A first theme was of the individual's efforts to strive to be 'good', as adjudicated by doctors. Coping was figured as an individual's task, with little reference to joint efforts with family members or healthcare professionals. Indeed, doctors loomed large in the imaginations of our participants, but were not sensed to be especially accessible. Family members and other healthcare professionals, whilst discussed in other contexts, were not generally characterised

For peer review only - http://bmjopen.bmj.com/site/about/guidelines.xhtml

as contributing to preventative coping efforts. The theme of individualised efforts to be 'good' aligns with other interview studies of survivors' rehabilitation, extending this observation to the context of secondary prevention. A second, novel theme was of reference by participants to 'personality' as a source of felt continuity and a resource for preventative coping. Appeals to essentialised notions of self may help survivors sustain feelings of biographical coherence, but may also prove an obstacle to behaviour change, as well as adaptive alterations in self-concept.

Contributorship statement: The study was conceived and designed by PSR, EN, FC, RM, JM and RD. PSR, FC, RM, JM and RD are academic researchers; EN was a student intern. Transcripts were coded by PSR and EN. PSR, RD and JM drafted the manuscript. All authors have read and approved the final manuscript.

Competing interests: None

Funding: This study is funded by the National Institute for Health Research's (NIHR) Programme Grant for Applied Research titled 'Developing primary care services for stroke survivors' (reference PTC-RP-PG-0213–20001). The views expressed are those of the authors and not necessarily those of the NIHR or the Department of Health and Social Care. JM is an

For peer review only - http://bmjopen.bmj.com/site/about/guidelines.xhtml

NIHR Senior Investigator.

Data sharing statement: Data are available upon reasonable request. Contact Jonathan Mant, jm677@medschl.cam.ac.uk

Ethics statement:

Favourable ethical opinion for the research was gained on 5th November 2015from East of England Cambridge South Research Ethics Committee IRAS project ID: 182927REC reference: 15/EE/0374.

For peer review only - http://bmjopen.bmj.com/site/about/guidelines.xhtml

References

1. Bhatnagar P, Wickramasinghe K, Wilkins E, Townsend N. Trends in the epidemiology of cardiovascular disease in the UK. Heart. 2016;102(24):1945-52.. https://doi.org/10.1136/heartjnl-2016-309573

2. Clarke P, Black SE. Quality of life following stroke: negotiating disability, identity, and resources. Journal of Applied Gerontology. 2005;24(4):319-36. https://doi.org/10.1177/0733464805277976

3. Crowfoot G, van der Riet P, Maguire J. Real-life experiences of people with transient ischaemic attack or minor stroke: A qualitative literature review. Journal of clinical nursing. 2018;27(7-8):1381-98. https://doi.org/10.1111/jocn.14271

4. Boger EJ, Demain SH, Latter SM. Stroke self-management: A focus group study to identify the factors influencing self-management following stroke. International Journal of Nursing Studies. 2015;52(1):175-87. https://doi.org/10.1016/j.ijnurstu.2014.05.006

5. Ellis J, Boger E, Latter S, Kennedy A, Jones F, Foster C, Demain S. Conceptualisation of the ‘good’self-manager: A qualitative investigation of stakeholder views on the self-management of long-term health conditions. Social

For peer review only - http://bmjopen.bmj.com/site/about/guidelines.xhtml

Science & Medicine. 2017;176:25-33. https://doi.org/10.1016/j.socscimed.2017.01.018

6. Fletcher S, Kulnik ST, Demain S, Jones F. The problem with self-management: Problematising self-management and power using a Foucauldian lens in the context of stroke care and rehabilitation. PloS one. 2019 Jun 19;14(6):e0218517. https://doi.org/10.1371/journal.pone.0218517
7. Vassilev I, Rogers A, Todorova E, Kennedy A, Roukova P. The articulation of neoliberalism: narratives of experience of chronic illness management in Bulgaria and the UK. Sociology of Health & Illness. 2017;39(3):349-64. https://doi.org/10.1111/1467-9566.12488
8. Abrahamson V, Wilson P. Positioning the six-month review in the recovery process post-stroke: The ideology of personal responsibility. Health & social care in the community. 2019;27(1):249-59. https://doi.org/10.1111/hsc.12677
9. Lazarus, R., Folkman, S. Stress, Appraisal and Coping. New York: Springer, 1984, p.34

For peer review only - http://bmjopen.bmj.com/site/about/guidelines.xhtml

10. Kuo BC. Collectivism and coping: Current theories, evidence, and measurements of collective coping. International Journal of Psychology. 2013;48(3):374-88. https://doi.org/10.1080/00207594.2011.640681

11. Vassilev I, Rogers A, Blickem C, Brooks H, Kapadia D, Kennedy A, Sanders C, Kirk S, Reeves D. Social networks, the 'work' and work force of chronic illness self-management: a survey analysis of personal communities. PloS one. 2013;8(4):e59723. https://doi.org/10.1371/journal.pone.0059723

12. Faircloth CA, Boylstein C, Rittman M, Young ME, Gubrium J. Sudden illness and biographical flow in narratives of stroke recovery. Sociology of health & illness. 2004;26(2):242-61. https://doi.org/10.1111/j.1467-9566.2004.00388.x

13. Simeone S, Savini S, Cohen MZ, Alvaro R, Vellone E. The experience of stroke survivors three months after being discharged home: A phenomenological investigation. European Journal of Cardiovascular Nursing. 2015;14(2):162-9. https://doi.org/10.1177/1474515114522886

14. Rochette A, Tribble DS, Desrosiers J, Bravo G, Bourget A. Adaptation and coping following a first stroke: a qualitative analysis of a phenomenological orientation.

For peer review only

International Journal of Rehabilitation Research. 2006;29(3):247-9. https://doi.org/10.1097/01.mrr.0000230051.24228.c8

15. Finch E, Foster M, Fleming J. Disrupted biographies: making sense of minor stroke after hospital discharge. Disability and Rehabilitation. 2020:1-8. https://doi.org/10.1080/09638288.2019.1708980

16. Carlsson GE, Möller A, Blomstrand C. Managing an everyday life of uncertainty–a qualitative study of coping in persons with mild stroke. Disability and Rehabilitation. 2009;31(10):773-82. https://doi.org/10.1080/09638280802638857

17. Dalvandi A, Heikkilä K, Maddah SS, Khankeh HR, Ekman SL. Life experiences after stroke among Iranian stroke survivors. International nursing review. 2010;57(2):247-53. https://doi.org/10.1111/j.1466-7657.2009.00786.x

18. Pallesen H. Body, coping and self-identity. A qualitative 5-year follow-up study of stroke. Disability and Rehabilitation. 2014;36(3):232-41. https://doi.org/10.3109/09638288.2013.788217

19. Persson HC, Törnbom K, Sunnerhagen KS, Törnbom M. Consequences and coping strategies six years after a subarachnoid hemorrhage–A qualitative study. PLoS One. 2017;12(8):e0181006. https://doi.org/10.1371/journal.pone.0181006

For peer review only - http://bmjopen.bmj.com/site/about/guidelines.xhtml

For peer review only

20. Jamison J, Graffy J, Mullis R, Mant J, Sutton S. Barriers to medication adherence for the secondary prevention of stroke: a qualitative interview study in primary care. British journal of general practice. 2016;66(649):e568-76. https://doi.org/10.3399/bjgp16X685609

21. Ravenhill G, Gkanasouli L, Lawrence M. Experiences of transient ischaemic attack diagnosis and secondary prevention: a qualitative review. British Journal of Neuroscience Nursing. 2019;15(5):S14-25. https://doi.org/10.12968/bjnn.2019.15.Sup5.S14

22. Parappilly BP, Mortenson WB, Field TS, Eng JJ. Exploring perceptions of stroke survivors and caregivers about secondary prevention: a longitudinal qualitative study. Disability and Rehabilitation. 2020;42(14):2020-6. https://doi.org/10.1080/09638288.2018.1544296

23. Schwarzer R, Knoll N. Positive coping: Mastering demands and searching for meaning. In S. J. Lopez & C. R. Snyder (Eds.), *Positive psychological assessment: A handbook of models and measures.* (pp. 393–409). American Psychological Association. 2003. https://doi.org/10.1037/10612-025

For peer review only - http://bmjopen.bmj.com/site/about/guidelines.xhtml

24. Intercollegiate Stroke Working Party. National Clinical Guidelines for Stroke, 5th Edition. Royal College of Physicians. 2016. https://www.rcplondon.ac.uk/guidelines-policy/stroke-guidelines

25. Braun V, Clarke V. Using thematic analysis in psychology. Qualitative Research in Psychology. 2006;3(2):77-101. https://doi.org/10.1191/1478088706qp063oa

26. Frith H, Gleeson K. Clothing and embodiment: Men managing body image and appearance. Psychology of Men & Masculinity. 2004;5(1):40. https://doi.org/10.1037/1524-9220.5.1.40

27. MacQueen, K. M., McLellan, E., Kay, K., & Milstein, B.. Codebook development for team-based qualitative analysis. *CAM Journal*, 1998;10(2):31–36. https://doi.org/10.1177/1525822X980100020301

28. Saldaña, J. (2015). *The coding manual for qualitative researchers*. London: Sage.

29. Croot EJ, Ryan TW, Read J, Campbell F, O'Cathain A, Venables G. Transient ischaemic attack: a qualitative study of the long term consequences for patients. BMC Fam Pract. 2014;15:174. https://doi.org/10.1186/s12875-014-0174-9

For peer review only - http://bmjopen.bmj.com/site/about/guidelines.xhtml

30. Morris R. Meeting the psychological needs of community-living stroke patients and carers: a study of third sector provision. Disability and Rehabilitation. 2016;38(1):52-61. https://doi.org/10.3109/09638288.2015.1014932

31. Playford ED, Siegert R, Levack W, Freeman J. Areas of consensus and controversy about goal setting in rehabilitation: a conference report. Clinical Rehabilitation. 2009;23(4):334-44. https://doi.org/10.1177/0269215509103506

32. Kidd L, Booth J, Lawrence M, Rowat A. Implementing supported self-management in community-based stroke care: a secondary analysis of nurses' perspectives. Journal of Clinical Medicine. 2020;9(4):985. https://doi.org/10.3390/jcm9040985

33. Foucault, M. (1985) The Use of Pleasure, trans. Robert Hurley, London: Penguin, p.26

34. Pound P, Gompertz P, Ebrahim S. Illness in the context of older age: the case of stroke. Sociology of health & illness. 1998;20(4):489-506. https://doi.org/10.1111/1467-9566.00112

35. Large R, Samuel V, Morris R. A changed reality: Experience of an acceptance and commitment therapy (ACT) group after stroke. Neuropsychological rehabilitation. 2019;30(8):1477-1496. https://doi.org/10.1080/09602011.2019.1589531

For peer review only - http://bmjopen.bmj.com/site/about/guidelines.xhtml

36. Hutton L, Ownsworth T. A qualitative investigation of sense of self and continuity in younger adults with stroke. Neuropsychological Rehabilitation. 2019;29(2):273-88. https://doi.org/10.1080/09602011.2017.1292922

37. Pickersgill M. How personality became treatable: The mutual constitution of clinical knowledge and mental health law. Social Studies of Science. 2013;43(1):30-53. https://doi.org/10.1177/0306312712457722

38. Higgs R, Soundy A. Factors which influence risk taking and the evolution of social-identity in stroke narratives: a thematic synthesis. Behavioral Sciences. 2020;10(2):46. https://doi.org/10.3390/bs10020046

39. Klevan T, Jonassen R, Sælør KT, Borg M. Using dyadic interviews to explore recovery as collaborative practices: challenging the epistemic norm of the single person perspective. International Journal of Qualitative Methods. 2020;19. https://doi.org/10.1177/1609406920967868

For peer review only - http://bmjopen.bmj.com/site/about/guidelines.xhtml

Standards for Reporting Qualitative Research

	topic	Page nos
1.	title	1
2.	Abstract	2
3.	Problem formulation	4-5
4.	Research Question	5
5.	Qualitative approach	6
6.	Researcher characteristics	9
7.	Context	6
8.	Sampling	6
9.	Ethical Issues	7
10.	Data collection methods	7-8
11.	Data collection instruments	6-8
12.	Units of study	7
13.	Data processing	8
14.	Data analysis	8-9
15.	Trustworthiness	8-9
16.	Synthesis	9-12
17.	Empirical data	9-12
18.	Integration with prior work	12-15
19.	Limitations	15-16
20.	Conflicts of interest	3,17
21.	funding	17

For peer review only
